# A Reliable Methodology for Determining Seed Viability by Using Hyperspectral Data from Two Sides of Wheat Seeds

**DOI:** 10.3390/s18030813

**Published:** 2018-03-08

**Authors:** Tingting Zhang, Wensong Wei, Bin Zhao, Ranran Wang, Mingliu Li, Liming Yang, Jianhua Wang, Qun Sun

**Affiliations:** 1 Department of Plant Genetics and Breeding, College of Agriculture, China Agricultural University/Beijing Key Laboratory of Crop Genetic Improvement/The Innovation Center (Beijing) of Crop Seed Sciences Ministry of Agriculture, Beijing 100193, China; ztt_cau@163.com (T.Z.); binzhaodave@outlook.com (B.Z.); 18763825710@163.com (R.W.); 13limingliu@cau.edu.cn (M.L.); wangjh63@163.com (J.W.); 2 National R&D Center for Agro-Processing Equipments, College of Engineering, China Agricultural University, Beijing 100083, China; weiwensong8@163.com; 3 College of Science, China Agricultural University, Beijing 100083, China; cauyanglm@163.com

**Keywords:** hyperspectral imaging, seed viability, dataset, PLS-DA, SVM

## Abstract

This study investigated the possibility of using visible and near-infrared (VIS/NIR) hyperspectral imaging techniques to discriminate viable and non-viable wheat seeds. Both sides of individual seeds were subjected to hyperspectral imaging (400–1000 nm) to acquire reflectance spectral data. Four spectral datasets, including the ventral groove side, reverse side, mean (the mean of two sides’ spectra of every seed), and mixture datasets (two sides’ spectra of every seed), were used to construct the models. Classification models, partial least squares discriminant analysis (PLS-DA), and support vector machines (SVM), coupled with some pre-processing methods and successive projections algorithm (SPA), were built for the identification of viable and non-viable seeds. Our results showed that the standard normal variate (SNV)-SPA-PLS-DA model had high classification accuracy for whole seeds (>85.2%) and for viable seeds (>89.5%), and that the prediction set was based on a mixed spectral dataset by only using 16 wavebands. After screening with this model, the final germination of the seed lot could be higher than 89.5%. Here, we develop a reliable methodology for predicting the viability of wheat seeds, showing that the VIS/NIR hyperspectral imaging is an accurate technique for the classification of viable and non-viable wheat seeds in a non-destructive manner.

## 1. Introduction

Seeds are the basis of the agricultural industry [1]. The viability of seeds is a critical factor for seed quality, which is closely related to resistance to biotic and abiotic stress, germination percentage, and plant performance [2], which decreases with an increasing storage period [3]. Wheat (*Triticum aestivum* L.) is one of the major food crops in the world [4]. An increased understanding of wheat seed viability would be beneficial to the wheat industry by ensuring a higher yield for farmers and reducing crop variability. Seed companies would also benefit from enhanced viability by being able to ensure a higher quality product. Traditional methods of determining the viability of wheat seeds according to the International Seed Testing Association (ISTA) rules include tetrazolium staining [5,6], conductivity tests [7], immunoassay tests, and germination tests [8,9,10]. However, they are labor-intensive, time-consuming, and destructive processes, which are subject to human error [9,11]. Therefore, non-destructive, highly sensitive methods must be developed to ensure the speed of measurement and the viability and germination of wheat seeds for the modern agro-industry.

With the advance of computer and optical sensor technology, various non-destructive testing technologies have been applied to detect seed vitality. However, each of them has its advantages and disadvantages and no single method can provide a definitive means of accurately measuring seed viability. For example, X-ray assessment of seeds only presents the structural integrity of seeds, but cannot directly identify whether seeds are dead [11,12], and does not scale to larger operations [2]. Machine vision systems, a type of artificial intelligence that deals with simulating human vision, can obtain the surface characteristics of seeds, such as shape, color, size, and external texture [13,14,15], but not the spectral information of the chemical composition, thus limiting the identification accuracy in detection [16,17]. Manickavasagan et al. applied a machine vision system to classify eight wheat classes by using monochrome images, and the classification accuracies were between 73% and 100% [18]. Near infrared spectroscopy (NIRS) can obtain chemical composition information, such as lipids, proteins, and carbohydrates, but is easily affected by the uniformity of sample distribution because it uses only one a single spot of samples [19]. Wu et al. found that NIRS was an effective technique to differentiate the varieties of Chinese cabbage seeds [20]. 

The hyperspectral imaging (HSI) technique [21,22], a combination of conventional imaging and spectroscopy, effectively overcomes these above shortcomings by providing both spatial and spectral information. In this system, spectral information, which is tightly linked to the chemical composition, is not only collected from a single point, but also from every pixel of an image [2,23]. The immense potential and advantage for the visible and near-infrared (VIS/NIR) and/or NIR hyperspectral imaging techniques has been demonstrated in a number of studies of different cereal grains, including corn [24,25], rice [26], wheat [27,28], and oats [29,30]. However, most of the work has focused on the classification of varieties [31,32], storage year [1], and geographical origins [33] of seeds, and there are few reports about discrimination on viable or non-viable grain seeds by hyperspectral imaging. 

Moreover, previous work on detecting seed viability based on spectroscopy testing technologies has focused on classification accuracy for germinating and non-germinating seeds, while the loss degree of viable seeds is often overlooked [10,12,34]. High classification accuracy for germination is not necessarily correlated with accuracy for viability. Low discrimination of viable seeds can lead to a loss of seed and an increase in cost, both of which limit productivity. 

Most studies conducted to date used germ-up side spectral data or mean spectral data of two sides to construct a model for discriminating the quality of seeds [16,34]. However, the influence of the scanning mode on the identified characteristics of the seeds is rarely considered. The scanning mode, which is suitable for distinguishing the viability of wheat seed, needs to be further explored. 

The purpose of this study was to explore the feasibility of using hyperspectral imaging technique for detecting the viability of the single wheat seed. The specific objectives were to (1) study whether spectral pre-processing methods can increase the accuracy of the classification models; (2) to identify the most effective wavelengths linked to seed viability; and, (3) to investigate the classification performance of the PLS-DA and SVM models to find a satisfactory combination of spectral dataset, pre-processing method, wavelengths, and classifier for the accurate discrimination of seed viability. 

## 2. Materials and Methods

### 2.1. Seed Preparation

Dry seeds of the wheat cultivar Dunmaiwang were purchased from a local market (Jinan, China) in October 2017. The moisture content of the seeds was 10.6%. A total of 160 samples were selected, which were similar in size and structurally intact. In this study, the accelerated aging test was implemented with all of the samples being set on a nylon mesh screen and suspended over 80 mL of water inside a plastic box (120 mm × 120 mm × 50 mm). The box was placed in an electronic oven, which was maintained at 41 °C and approximately 100% relative humidity [35]. After four days, the seeds were removed and were subject to hyperspectral imaging and germination assessment until they naturally dried to their original moisture content. Thus, we were able to directly associate reflectance data from each seed with its germination status (yes = 1 or no = 2) [12]. 

### 2.2. Hyperspectral Imaging System

**Figure 1:**
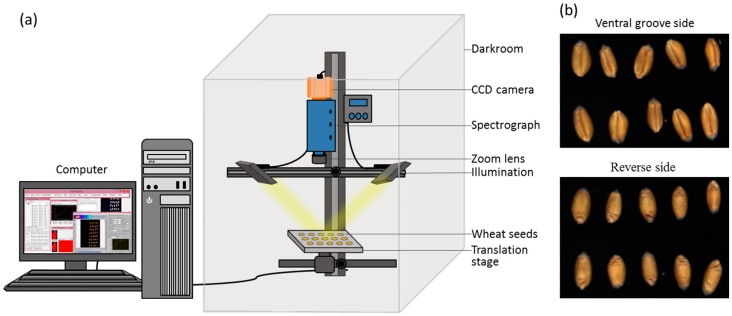
(**a**) Schematic diagram of the hyperspectral imaging system. (**b**) Hyperspectral images of the ventral groove and reverse side of wheat seeds.

An assembled hyperspectral imaging system covering the range of 400–1000 nm was applied to acquire images of wheat seeds (Figure 1). The system was composed of a imaging spectrograph (Imspector V10E; Spectral Imaging Ltd., Oulu, Finland), a charge-coupled device (CCD), camera (EM285CL; Raptor Photonics, Ltd., Ireland, UK), zoom lens (OLE23; Schneider, Germany), two 150 W tungsten-halogen lamps (IT 3900 e; Illumination Technologies Inc., New York, NY, USA) for illumination, a translation stage driven by a stepper motor (IRCP0076-1COMB; Isuzu Optics Corp., Hsinchu, Taiwan), data acquisition software (Spectral Image software; Isuzu Optics Corp., Hsinchu, Taiwan), a computer, and a darkroom. The data acquisition software could set the speed of the motor, exposure time, and wavelength range.

### 2.3. Image Acquisition and Calibration

Each wheat seed, both ventral groove and reverse sides, was placed on the translation stage and transmitted to the camera to be scanned line by line at 1.3 mm/s with a 15 ms exposure time to acquire three-dimensional (*x*, *y*, *λ*) hyperspectral images. These images included the linear array scanning by the detector along the *y*-direction and the movement of the sample in the *x*-direction. In this study, the images were acquired with 1004 pixels in the *x* direction, 1662 pixels in the *y* direction, and 766 wavelengths in the *λ*-direction, respectively. During image acquisition in the laboratory, relative humidity was 30%, and the temperature was 24 °C. The image acquisition was conducted with Spectral Image software. For calculating the reflectance spectrum, the spectral raw images (*I*_0_) of the wheat seeds were calibrated using two reference standards: the “white” one (*W*) was acquired with a standard white Teflon tile to establish the maximum reflectance conditions, and the “black” one (*B*) obtained by turning off the light source together with covering the camera lens with its opaque cap to define the no-reflectance condition. Then, the calibrated image (*I*) was calculated according to the following formula [36]:

$$I=\frac{I_{0}-B}{W-B}$$

The image calibrations were conducted using HSI Analyzer (Isuzu Optics Corp, Hsinchu, Taiwan).

### 2.4. Germination Assessment

After HSI spectra collection of samples of both sides of the wheat seeds, a germination test was implemented to check for seed viability [37]. In brief, each seed was placed in a plastic container (120 × 120 × 50 mm) on a germination paper (Whatman Paper, Whatman International, Maidstone, UK), which was kept moist by submerging two edges in water. A Petri dish (9 cm) was used to secure the seed and the germination paper. After seven days at 25 ± 1 °C with continuous light, germination counts were conducted. We recorded a seed as germinated (yes = 1) if the plumule and radicle were both over 2 mm long, and non-germinated (no = 2) if not. 

### 2.5. Spectral Data Extraction

After the images were corrected, the background of every image was removed, according to the contrast of the relative reflectance intensity. Then, a series of steps was carried out to extract the spectral data. First, the beginning and ending ranges were omitted from every hyperspectral data file, as they were greatly affected by stochastic noise. Therefore, 688 spectral bands from 430 nm to 970 nm were chosen for further analysis. Second, wheat seeds were segmented by a threshold imaging procedure at 830 nm to create a mask of the region of interest (ROI). The spectra of each pixel from every wheat seed was extracted and averaged. The image segmentation and spectral data extraction were conducted by using ENVI 5.1 (ITT Visual Information Solutions, Boulder, CO, USA).

### 2.6. Spectra Preprocessing

**Figure 2:**
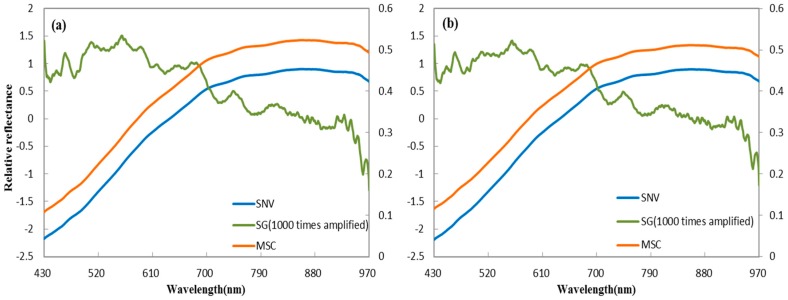
Average spectra preprocessed by standard normal variate (SNV), Savitzky-Golay (SG), and multiplicative scatter correction (MSC) methods; (**a**) ventral groove side; and, (**b**) reverse side.

Spectral noise (e.g., the effects of the operation, instrument, and the environment) is unavoidable in collecting hyperspectral images of the sample, and can affect later analysis. This interference information in the spectral curve decreases the signal: noise ratio and reduces the usefulness of the spectrum data. Extraction of ROIs can improve the data, and pre-processing methods can further maximize usefulness [38,39]. Various pre-processing methods are currently available, such as multiplicative scatter correction (MSC) [40,41], standard normal variate (SNV) [42,43], Savitzky-Golay (SG), and its derivate [16]. MSC and SNV are used to account for additive/multiplicative and scatter effects, respectively, in spectral data, and both can be used in combination with other measures to improve the signal to noise ratio. Savitzky-Golay is used to expose valuable information latent in a spectrum, with the Savitzky-Golay derivative as a frequently used method. It is important to choose an appropriate method for future analysis. In this study, before selecting optimal wavelength, the spectral data were separately preprocessed by the standard normal variate (SNV), multiplicative scatter correction (MSC), and derivatives that are based on the Savitzky-Golay algorithm with a gap of seven points (Figure 2). 

### 2.7. Optimal Wavelength Selection

The high-dimensional hyperspectral data, comprised of many congruent wavelengths, suffered from multicollinearity and redundancy. Optimal wavelength selection can select the effective wavelengths, which greatly accelerates the computation speed, leading to a time-saving calibration process, and can improve the modelling accuracy. Therefore, optimal wavelength selection is widely applied. The successive projections algorithm (SPA) is a forward selection algorithm that is regarded as an effective waveband selection method [44]. Previous research showed that it could minimize the collinearity among variables effectively [32,45]. SPA contains three main steps. Initially, candidate variables are selected according to the maximum projection value on the columns of the spectral matrix [46]. Then, all of the selected variables are evaluated by root mean square error (RMSE). Finally, the variables, which are irrelevant to the properties being predicted, are deleted [45]. The above procedures for SPA were applied in MATLAB R2014a (The Math Works, Natick, MA, USA).

### 2.8. Development of Classification Models

Choosing a reliable classifier is a significant step in building a classification model. Partial least square discriminant analysis (PLS-DA) [47,48] and support vector machines (SVM) [12,49] are widely-used classifiers. SVM is especially suitable for small datasets with high-dimensional feature spaces. In this study, classification models of different viability of wheat seeds were built by SVM and PLS-DA. In the process of the SVM algorithm, the C-Support Vector Classification (C-SVC) and radial basis function (RBF) kernel were selected. In addition, the grid-search and five-fold cross-validation were used to optimize the parameters. The leave-one-out method was applied for the PLS-DA algorithm [16]. In the processing of PLS-DA, the number of latent variables varied, while the correct percentage of seeds were maximized and held constant in one PLS-DA model. Each spectral dataset was divided into two groups, 106 wheat seeds as calibration sets (75 germinating and 31 non-germinating) and 54 seeds as prediction sets (38 germinating and 16 non-germinating). For the mixture dataset, the data from both sides of 106 seeds were used as calibration sets, and different proportions of data from the ventral groove and reserve sides of seeds were used for the prediction set in the models (Table 1).

Models were judged by four indices, which were overall accuracy (accuracy), viability accuracy (recall), final germination percentage (precision), and F-measure. In addition, the initial germination percentage of the prediction set was 70.4%. All of the steps that are involved in the SVM and PLS-DA were implemented in MATLAB R2014a (The Math Works, Natick, MA, USA).

The overall accuracy (classification accuracy of all seeds), viability accuracy (classification accuracy of viable seeds), final germination percentage, and F-measure were calculated according to the following formula:

$$\mathrm{Overall}\;\mathrm{accuracy}\left(\mathrm{Accuracy}\right)=\frac{G+NG}{G+NG+Gr+NGr}$$

$$\mathrm{Viablity}\;\mathrm{accuracy}\left(\mathrm{Recall}\right)=\frac{G}{G+Gr}$$

$$\mathrm{Final}\;\mathrm{germination}\;\mathrm{percentage}\left(\mathrm{Precision}\right)=\frac{G}{G+NGr}$$

$$F-\mathrm{measure}=2\times \frac{Recall\times Precision}{Recall+Precision}$$

where *G*, is the number of germinating seeds predicted correctly by the model; *NG* is the number of non-germinating seeds predicted correctly by the model; *Gr* is the actual number of germinating seeds incorrectly regarded as non-germinating seeds by the model; and, *NGr* is the actual number of non-germinating seeds that were incorrectly regarded as germinating seeds by the model. 

## 3. Results

### 3.1. Spectral Characteristics

**Figure 3:**
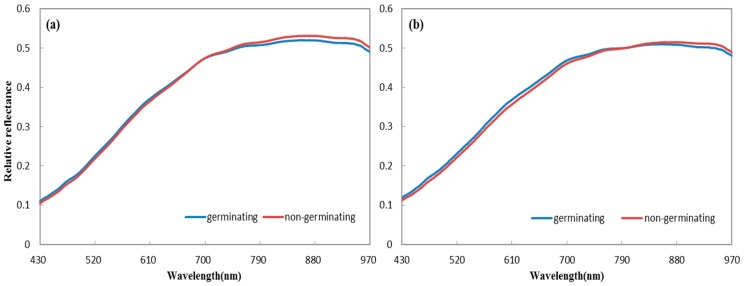
Average spectra of germination and non-germination seeds for (**a**) the ventral groove side and (**b**) the reverse side.

Hyperspectral information from both sides of wheat seeds was acquired (Figure 3). Obvious spectral differences were observed between germinating and non-germinating seeds in the average spectra of the two sides. For the ventral groove side, the values of average spectra of germinating seeds were higher at the wavelengths of 430–691 nm, but lower at 692–980 nm, when compared with those of non-germinating seeds. The average spectra of reverse side showed a similar trend. Germinating seeds showed higher average spectra at 430–799 nm, while non-germinating seeds exhibited higher average spectra at 800–980 nm. These differences indicate that hyperspectral features are influenced by seed vitality and that useful information exists in the hyperspectral imaging of both sides for seed classification.

### 3.2. Optimal Wavelengths Selected by the SPA Algorithm

After preprocessing, the optimal wavelengths were chosen by SPA, which is a novel method to minimize the collinearity among wavebands and select the optimal wavebands. The root mean square error (RMSE) was calculated to select the optimal number of wavebands when operating the SPA. The optimal wavelengths were acquired on the minimum RMSE with no overfitting problem (Table 2). Nearly all of the selected wavelengths were located near particular regions of the spectral range, which indicates some connection between seed reflectance and chemical information. For instance, many selected bands were located near the absorbance bands of plant pigments peaks: carotenoids (448, 471 nm), chlorophyll a (430, 662, and 680 nm), and chlorophyll b (448, 642 nm) [50]. In addition, bands near 970 nm may correspond to the O–H stretching (2nd overtone) and C–H stretching (3rd overtone) [33,50]. Bands that exceed 760 nm were disproportionately represented in the selected wavelengths of the reverse side. This could be due to the reverse side containing the embryo with more oil, moisture, and other compounds. Accordingly, the reflectance in narrow wavebands associated with chemical information may be the most useful to classify seed germination.

### 3.3. Classification by SVM and PLS-DA

After using the SPA method to select the spectral wavelength of different spectral datasets, the full wavelength data, as well as the selected wavelength data, were analyzed by PLS-DA and SVM to establish classification models. The results for the ventral groove side spectral dataset are shown in Table 3 and Supplemental Table S1. The performance for PLS-DA and SVM models based on pre-processed spectral data were better than those that were based on the raw spectral data. In all cases, the PLS-DA classification model combining the SNV and SPA methods obtained the most accurate results, with an overall correct discrimination rate for the prediction set of 85.2%, where the viability accuracy and F-measure were 89.5%. After SNV-SPA-PLS-DA model selection, the germination percentage of this seed lot increased from 70.4% to 89.5% (Table 3). 

In the case of the reverse-side spectral dataset, PLS-DA and SVM models based on raw spectral data obtained better performance than that of the ventral groove side (Table 3 and Supplemental Table S1). All of the pre-processing methods combined with the SPA method could effectively optimize the performance for PLS-DA and SVM models. In these cases, the SVM classification model based on optimum wavelength data with SG method exhibited a highest classification capability with an overall accuracy of 88.9%, where the viability accuracy and F-measure were 97.4% and 92.5%, respectively. This model could increase the germination percentage from 70.4% to 88.1% (Table 3).

Models based on the hyperspectral data of the ventral groove and reverse sides indicated that there were useful spectral data in both sides for identifying viable wheat seeds. Therefore, we further investigated the discrimination ability of mean and mixture spectra from both sides. Analyzing the performance of the models based on mean spectra, we found that SNV-SPA-PLS-DA was the optimal method for seed classification. This model showed high discrimination ability with an overall classification accuracy of 87.0%, a viability accuracy of 89.5%, and with an F-measure of 90.7% in the prediction set. This model could promote the germination percentage of the seed lot from 70.4% to 91.9% (Table 3 and Supplemental Table S2). Our results indicated that the model based on the mean spectral dataset showed a lower classification accuracy of overall seeds and viable seeds than that based on the reverse spectral dataset. However, the models using mean spectrum data exhibited a higher ability for improving the final germination percentage of the seed lot than did the optimal model using the reverse side spectrum data.

For modeling that is based on the mixture spectral dataset, the calibration and prediction sets were comprised of equal proportions of the ventral groove side and reverse side data, as described in Table 1. The best performance model based on the mixture dataset was generated by combining SNV with SPA, and this SNV-SPA-PLS-DA model had a high classification accuracy rate for whole seeds (88.9%) and maintained a relatively high viability accuracy and F-measure (92.1%, 92.1%) in the prediction set. After screening by this model, the average final germination percentage of the seed lot reached 92.1%, which was higher than the results of models using the reverse side and mean spectrum (Table 3 and Supplemental Table S3).

To further analyze the practical modeling effect of the SNV-SPA-PLS-DA model, different proportions of seeds comprising the ventral groove side and reverse side for the prediction set were put into this model (Table 1). Interestingly, the models performed better with an increasing reverse side ratio, while models performed less accurately with an increase in the number of ventral groove sides (Table 4). However, even when the prediction set contained 100% ventral groove sides, the model could still improve the germination percentage of the seed lot to 89.5%, and effectively recognize the viable and nonviable wheat seeds.

## 4. Discussion

To select the best pre-processing methods for reducing noise interference in the spectral data and to enhance model accuracy, different preprocessing methods were applied to investigate the model performance. The results showed that the SG, SNV, and MSC methods had different effects on the performance of the models that are based on different spectral datasets. Among them, the SG and SNV methods were more appropriate than MSC to preprocess the spectral information of four spectral datasets (Table 3). Thus, models must be constructed to employ specific preprocessing methods for different datasets [25]. In addition, our results indicated that the SPA algorithm was suitable for selecting the important variables and for modeling the viability screening of wheat seeds. Constructing the model using the particular wavelengths selected by SPA reduced hyperspectral imaging and processing time [33,51]. Most optimum models that were based on different spectral datasets were produced by using pre-processing methods and particular wavelengths selected by SPA. SVM, and PLS-DA are widely used to classify the varieties and storing-years for seeds of different species [52,53]. Our work indicated that these two classifiers could distinguish the viability of wheat seeds. 

Overall classification accuracy is a weighted average of viability and non-viability accuracy, which is influenced by both the accuracy of determining the viability and non-viability, and by the frequency of viable and non-viable seeds. In this work, the original germination percentage of the seed lot was 70.4%, suggesting that viability accuracy carried more weight in overall accuracy. Therefore, high overall accuracy cannot completely reflect the performance of the models. For example, the SG-SPA-SVM model based on reverse spectra showed higher overall classification accuracy, but a lower ability for improving the final germination percentage of the seed lot than did the SNV-SPA-PLS-DA model using the ventral groove side spectrum data. When selecting a practical model for seed viability classification, not only the overall accuracy, but also the accuracy of viable seeds and the enhancing effect of final germination percentage should be considered as selection criteria. Among the models that are based on four datasets, in each of which germination of the seed lot could be increased to more than 89%, the SNV-SPA-PLS-DA model based on the mixture spectra obtained the highest classification accuracy of whole and viable seeds (Table 3). In this model, high overall accuracy and viable seeds were obtained by only using selected wavelengths. This system greatly reduced the number of used wavelengths from 688 to 16, and could be applied to develop multispectral screening machines for seed companies and farmers. In addition to providing high classification capability, it is cost-effective, requiring only one scanner to acquire spectral information. Acquisition of average spectral data requires two cameras to simultaneously screen with a specific machine that is needed to keep the seeds in the appropriate orientation for reverse-side information acquisition, negatively affecting the speed and cost of production. 

## 5. Conclusions

The methodology of hyperspectral image acquisition, pre-processing, and of wavelength and classifier selection was established for the classification of viable and non-viable wheat seeds. SNV and SG preprocessing are more appropriate than MSC preprocessing. Likewise, the SPA method, which extracted the most effective wavebands and optimized models, was suitable for wheat seed characterization according to viability. Both SVM and PLS-DA algorithms could be used for modeling to predict the viability of wheat seeds. Our results showed that the SNV-SPA-PLS-DA model that is based on the mixture dataset had a great classification capability, with an overall accuracy of more than 85.2%, with a viability accuracy and F-measure of more than 89.5%, in the prediction set. Furthermore, after screening by this model, the average final germination percentage of the seed lot could exceed 89.5%. Finally, we conclude that hyperspectral imaging spectroscopy has great potential to effectively differentiate the viability of wheat seeds in the VIS/NIR range (400–1000 nm).

## Figures and Tables

**Table 1:** Different proportions of the ventral groove side and the reverse side for constructing the calibration set and prediction set.

No.	Calibration Set		Prediction Set	
	Ventral Groove	Reverse	Ventral Groove	Reverse
1	106	106	27	27
2	106	106	0	27
3	106	106	13	27
4	106	106	27	13
5	106	106	27	0

**Table 2:** Selected wavelengths by successive projections algorithm (SPA).

Dataset	Pre-Processing	Selected Wavelengths (nm)
Ventral groove	RAW	430 442 489 516 538 591 652 673 692 777 815 940 959 968
	SG	431 462 490 505 959 969 970
	SNV	431 432 438 439 449 450 475 491 521 538 554 606 673 777 847 910 932 937 945 965 968 968
	MSC	430 438 444 493 521 554 591 675 696 810 906
Reverse	RAW	431 436 445 465 493 554 622 670 745 819 880 915 959 965
	SG	434 438 462 485 525 825 891 969 970
	SNV	430 432 453 474 494 523 574 673 745 773 853 917 958 961 965
	MSC	431 434 445 448 452 494 554 591 669 696 810 839 881 908 915 958 965
Mean	RAW	430 431 432 438 454 488 529 554 600 640 666 714 749 777 836 881 901 949 961 968
	SG	431 434 438 442 446 461 485 504 548 597 681 862 886 908 943 956 959 966 969 970
	SNV	431 438 445 491 582 641 672 722 839 881 908 931 957 965
	MSC	432 438 444 491 521 554 591 672 745 810 881 901 957 965
Mixture	RAW	430 489 558 653 814 934
	SG	430 431 448 490 505 959 969 970
	SNV	432 471 494 518 533 550 675 756 774 783 792 804 808 831 948 968
	MSC	430 467 493 645 961

**Table 3:** The best results of models based on each spectral dataset.

Datasets	Pre-Processing	No. of Wavelengths	Models	Calibration Set	Prediction Set			
				Overall Accuracy (%)	Overall Accuracy (%)	Viability Accuracy (%)	Final Germination Percentage (%)	F-Measure (%)
Ventral groove	SNV	^a^ S(22)	PLS-DA	85.8	85.2	89.5	89.5	89.5
Reverse	SG	S(9)	SVM	89.6	88.9	97.4	88.1	92.5
Mean	SNV	S(14)	PLS-DA	87.7	87	89.5	91.9	90.7
Mixture	SNV	S(16)	PLS-DA	90.1	88.9	92.1	92.1	92.1

^a^ S represents selected wavelengths.

**Table 4:** Results of the models based on different proportions of the ventral groove side and reverse side.

No.	Calibration Set	Prediction Set			
	Overall Accuracy (%)	Overall Accuracy (%)	Viability Accuracy (%)	Final Germination Percentage (%)	F-Measure (%)
1	90.1	88.9	92.1	92.1	92.1
2	90.1	92.6	94.7	94.7	94.7
3	90.1	90.0	96.6	90.3	93.3
4	90.1	87.5	93.1	90.0	91.5
5	90.1	85.2	89.5	89.5	89.5

## Supplementary Materials

The supplementary materials are available online at http://www.mdpi.com/1424-8220/18/3/813/s1. Table S1. Performance of PLS-DA and SVM models based on the ventral groove and reverse spectral datasets. Table S2. Performance of PLS-DA and SVM models based on the mean spectral dataset. Table S3. Performance of PLS-DA and SVM models based on a mixed spectral dataset.
