# Supplementary material for: A Reliable Methodology for Determining Seed Viability by Using Hyperspectral Data from Two Sides of Wheat Seeds
Source: Sensors (Basel). 2018 Mar 8;18(3):813. doi: 10.3390/s18030813 (PMC5876662; doi:10.3390/s18030813)
Supplement: Supplementary file 1 [file sensors-18-00813-s001.pdf]

Article

A Reliable Methodology for Determining Seed Viability by Using Hyperspectral Data from Two Sides of Wheat Seeds

Tingting Zhang [1,†], Wensong Wei [2,†], Bin Zhao1 [1], Ranran Wang [1], Mingliu Li [1], Liming Yang [3], Jianhua Wang [1] and Qun Sun [1,*]

[1] Department of Plant Genetics and Breeding, College of Agriculture, China Agricultural University/Beijing Key Laboratory of Crop Genetic Improvement/The Innovation Center (Beijing) of Crop Seed Sciences Ministry of Agriculture, Beijing 100193, China; ztt_cau@163.com(T.Z.); binzhaodave@outlook.com(B.Z.); 18763825710@163.com(R.W.); 13limingliu@cau.edu.cn(M.L.); wangjh63@163.com(J.W.)

[2] National R&D Center for Agro-Processing Equipments, College of Engineering, China Agricultural University, Beijing 100083, China; weiwensong8@163.com

[3] College of Science, China Agricultural University, Beijing 100083, China; cauyanglm@163.com

* Correspondence: sqcau@126.com; Tel.: +86-10-6273-2775

† These authors contributed equally to this work.

Supplementary data

Supplemental Table S1. Performance of PLS-DA and SVM models based on the ventral groove and reverse spectral datasets.

Datasets	Pre-processing	No. of wavelengths	Models	Calibration set	Prediction set			
				Overall accuracy (%)	Overall accuracy (%)	Viability accuracy (%)	Final germination percentage (%)	F-measure (%)
Ventral groove	RAW	[b] F(688)	PLS-DA	77.4	68.5	73.7	80.0	76.7
			SVM	75.5	74.1	100.0	73.1	84.5
		[a] S(14)	PLS-DA	76.4	75.9	84.2	82.1	83.1
			SVM	75.5	75.9	100.0	74.5	85.4
	SG	F(688)	PLS-DA	72.6	74.1	86.8	78.6	82.5
			SVM	86.8	83.3	94.7	83.7	88.9
		S(7)	PLS-DA	76.4	83.3	89.5	87.2	88.3
			SVM	77.4	74.1	100.0	73.1	84.5
	SNV	F(688)	PLS-DA	73.6	72.2	81.6	79.5	80.5
			SVM	85.8	77.8	94.7	78.3	85.7
		S(22)	PLS-DA	85.8	85.2	89.5	89.5	89.5
			SVM	87.5	70.4	100.0	70.4	82.6
	MSC	F(688)	PLS-DA	73.6	72.2	81.6	79.5	80.5
			SVM	85.8	77.8	92.1	79.5	85.3
		S(11)	PLS-DA	83.0	81.5	89.5	85.0	87.2

			SVM	75.5	79.6	100.0	77.6	87.4
Reverse	RAW	F(688)	PLS-DA	84.0	79.6	86.8	84.6	85.7
			SVM	81.1	79.6	94.7	80.0	86.7
		S(14)	PLS-DA	85.8	85.2	84.2	94.1	88.9
			SVM	84.0	77.8	84.2	84.2	84.2
	SG	F(688)	PLS-DA	85.8	85.2	86.8	91.7	89.2
			SVM	87.7	87.0	94.7	87.8	91.1
		S(9)	PLS-DA	87.7	87.0	89.5	91.9	90.7
			SVM	89.6	88.9	97.4	88.1	92.5
	SNV	F(688)	PLS-DA	87.7	85.2	89.5	89.5	89.5
			SVM	85.8	85.2	100.0	82.6	90.5
		S(15)	PLS-DA	90.0	87.0	89.5	91.9	90.7
			SVM	86.8	87.0	97.4	86.0	91.3
	MSC	F(688)	PLS-DA	84.0	83.3	94.7	83.7	88.9
			SVM	87.7	87.0	97.4	86.0	91.3
		S(17)	PLS-DA	87.7	85.2	89.5	89.5	89.5
			SVM	90.6	83.3	100.0	80.9	89.4

[a] S represents selected wavelengths. [b] F represents full wavelengths.

Supplemental Table S2. Performance of PLS-DA and SVM models based on the mean spectral dataset.

Datasets	Pre-processing	No. of wavelengths	Models	Calibration set	Prediction set			
				Overall accuracy (%)	Overall accuracy (%)	Viability accuracy (%)	Final germination percentage (%)	F-measure (%)
Mean	RAW	[b] F(688)	PLS-DA	83.0	83.3	89.5	87.2	88.3
			SVM	80.2	74.1	94.7	75.0	83.7
		[a] S(20)	PLS-DA	84.0	85.2	84.2	94.1	88.9
			SVM	78.3	72.2	97.4	72.5	83.1
	SG	F(688)	PLS-DA	84.9	81.5	89.5	85.0	87.2
			SVM	88.7	87.0	97.4	86.0	91.3
		S(20)	PLS-DA	85.8	83.3	86.8	89.2	88.0
			SVM	88.7	83.3	94.7	83.7	88.9
	SNV	F(688)	PLS-DA	83.0	81.5	92.1	83.3	87.5
			SVM	87.7	85.2	100.0	82.6	90.5
		S(14)	PLS-DA	87.7	87.0	89.5	91.9	90.7
			SVM	85.8	85.2	100.0	85.2	92.0
	MSC	F(688)	PLS-DA	80.2	81.5	92.1	83.3	87.5
			SVM	86.8	85.2	94.7	85.7	90.0

		S(14)	PLS-DA	85.8	85.2	92.1	87.5	89.7
			SVM	87.7	87.0	97.3	85.7	91.1

[a] S represents selected wavelengths. [b] F represents full wavelengths.

Supplemental Table S3. Performance of PLS-DA and SVM models based on a mixed spectral dataset.

Datasets	Pre-processing	No. of wavelengths	Models	Calibration set	Prediction set			
				Overall accuracy (%)	Overall accuracy (%)	Viability accuracy (%)	Final germination percentage (%)	F-measure (%)
Mixture	RAW	[b] F(688)	PLS-DA	86.3	85.2	89.5	89.5	89.5
			SVM	82.1	77.8	97.4	77.1	86.1
		[a] S(6)	PLS-DA	76.4	75.9	84.2	82.1	83.1
			SVM	77.4	77.8	92.1	79.5	85.3
	SG	F(688)	PLS-DA	92.0	83.3	86.8	89.2	88.0
			SVM	88.7	87.0	94.7	87.8	91.1
		S(8)	PLS-DA	79.7	79.6	86.8	84.6	85.7
			SVM	82.5	81.5	94.7	81.8	87.8
	SNV	F(688)	PLS-DA	92.9	87.0	89.5	91.9	90.7
			SVM	88.7	88.9	97.4	86.0	91.3
		S(16)	PLS-DA	90.1	88.9	92.1	92.1	92.1
			SVM	89.2	83.3	97.4	82.2	89.2
	MSC	F(688)	PLS-DA	91.0	85.2	86.8	91.7	89.2
			SVM	88.2	87.0	94.7	87.8	91.1
		S(5)	PLS-DA	79.7	85.2	92.1	87.5	89.7
			SVM	83.5	83.3	94.7	83.7	88.9

[a] S represents selected wavelengths. [b] F represents full wavelengths.
